# Supplementary material for: Prevalence of gastroesophageal reflux disease and its associated risk factors among the adult population of Mthatha, Eastern Cape, South Africa
Source: PLoS One. 2025 Dec 1;20(12):e0337059. doi: 10.1371/journal.pone.0337059 (PMC12668490; doi:10.1371/journal.pone.0337059)
Supplement: Attachment — Submitted filename: Response to Reviewers 21102025.docx [file pone.0337059.s003.docx]

Authors’ Note:

Dear reviewers,

Greetings and thank you for all the additional comments to strengthen the quality of the manuscript.

Please find the responses to the comments. Thank you

Reviewer #1: Dear Authors, All the comments have not been addressed. Here are the comments that need to be addressed:

1. Line 123 – “… selected using a sampling constant of six”, how was this interval calculated.

**Response:** Thank you for this comment. The interval was calculated by dividing the population size (N= 2500) by the estimated sample size (n=406) ~6.2. Please verify if Line 126 to Line 128 of the manuscript addresses this comment.

2. Reviewer #1: How did you use systematic sampling?

**Response:** Thank you for requesting clarity on this important component on systematic sampling. In this study, it began with a random selection of the first participant on each data collection day, then every sixth person. Please verify if Line 125 to Line 126 of the manuscript addresses this comment.

Reviewer #1: What was the sampling frame?

**Response:** Thank you for requesting clarity on this important component on systematic sampling. The sampling frame was the total number of people who will be available (expected to visit the outpatient department of the hospital) during the one-month data collection period (N= 2500). Please verify if Line 123 to Line 125 of the manuscript addresses this comment.

Reviewer #1: 3. Please revise the result and discussion part.

**Response:** Thank you for highlighting the need to revise the results and discussion part. This has been done. Please refer to Table 3 and Line 239.

Reviewer #3: My earlier concerns regarding unclear inclusion and exclusion criteria, the absence of clarity around comorbidities, and the need to better acknowledge potential confounding factors have been adequately addressed in the revised manuscript. The authors have clarified the study population, defined comorbidities, acknowledged the limitations around unmeasured variables such as anti-hypertensive drug use, and re-analyzed the data to focus appropriately on Mthatha. These revisions have improved the methodological transparency and strengthened the manuscript.

**Response:** Thank you. We highly appreciate your comment.

Reviewer #4: I am satisfied with the revisions made by the authors. The data underlying the findings have been made fully available, and the manuscript is clearly presented in standard English. The work is technically sound, with the data appropriately supporting the conclusions. Furthermore, the statistical analyses have been conducted rigorously and appropriately. I therefore find the manuscript suitable for acceptance.

**Response:** Thank you. We highly appreciate your comment.

Reviewer #6: The manuscript in its current form is suitable for publication as all comments have been addressed. However, please replace the symbol ‘@’ in the Table 3 with a clearer notation such as ‘N\C’ (Not Computed). For clarity, you may also include a footnote below the table, for example:
Note: N\C = Not Computed.

**Response:** Thank you for this quality improvement recommendation for the results section. The recommended input was considered. Please refer to Table 3 and Line 239.
